# Integrated Anaerobic-Aerobic Biodegradation of Multiple Contaminants Including Chlorinated Ethylenes, Benzene, Toluene, and Dichloromethane

**DOI:** 10.1007/s11270-016-3216-1

**Published:** 2016-12-14

**Authors:** Miho Yoshikawa, Ming Zhang, Koki Toyota

**Affiliations:** 1 Geological Survey of Japan, National Institute of Advanced Industrial Science and Technology (AIST), 1-1-1, Higashi, Tsukuba, Ibaraki 305-8567 Japan; 2 Graduate School of Bio-Applications and Systems Engineering, Tokyo University of Agriculture and Technology, 2-24-16, Nakacho, Koganei, Tokyo Japan

**Keywords:** Bioremediation, Multiple contaminants, Chlorinated ethylenes, Benzene, Integrated anaerobic and aerobic biodegradation, *Dehalococcoides*

## Abstract

Complete bioremediation of soils containing multiple volatile organic compounds (VOCs) remains a challenge. To explore the possibility of complete bioremediation through integrated anaerobic-aerobic biodegradation, laboratory feasibility tests followed by alternate anaerobic-aerobic and aerobic-anaerobic biodegradation tests were performed. Chlorinated ethylenes, including tetrachloroethylene (PCE), trichloroethylene (TCE), *cis*-dichloroethylene (*cis*-DCE), and vinyl chloride (VC), and dichloromethane (DCM) were used for anaerobic biodegradation, whereas benzene, toluene, and DCM were used for aerobic biodegradation tests. Microbial communities involved in the biodegradation tests were analyzed to characterize the major bacteria that may contribute to biodegradation. The results demonstrated that integrated anaerobic-aerobic biodegradation was capable of completely degrading the seven VOCs with initial concentration of each VOC less than 30 mg/L. Benzene and toluene were degraded within 8 days, and DCM was degraded within 20 to 27 days under aerobic conditions when initial oxygen concentrations in the headspaces of test bottles were set to 5.3% and 21.0%. *Dehalococcoides* sp., generally considered sensitive to oxygen, survived aerobic conditions for 28 days and was activated during the subsequent anaerobic biodegradation. However, degradation of *cis*-DCE was suppressed after oxygen exposure for more than 201 days, suggesting the loss of viability of *Dehalococcoides* sp., as they are the only known anaerobic bacteria that can completely biodegrade chlorinated ethylenes to ethylene. Anaerobic degradation of DCM following previous aerobic degradation was complete, and yet-unknown microbes may be involved in the process. The findings may provide a scientific and practical basis for the complete bioremediation of multiple contaminants in situ and a subject for further exploration.

## Introduction

Bioremediation of soils containing multiple contaminants remains a challenge in environmental science and engineering because complete biodegradation of all components is necessary but very difficult to accomplish (Low et al. 2007). At many contaminated sites, volatile organic compounds (VOCs), especially chlorinated ethylenes and benzene, are the most common contaminants (US EPA 2013a; Ministry of the Environment, Japan 2014), with multiple VOCs coexisting in some cases. Contamination with VOCs is typically characterized as being nonhomogeneous in its distribution and concentration and spreading over a large area (US EPA 2000), and bioremediation is typically applicable to such sites. Although bioremediation has been widely applied to sites contaminated with a single VOC or several similar VOCs, such as chlorinated ethylenes, application of bioremediation to sites contaminated with multiple contaminants is considered difficult (Vidali 2001).

Conditions for bioremediation of chlorinated ethylenes and benzene differ, which makes complete remediation of multiple VOCs using a single approach difficult. In general, anaerobic bioremediation is applicable to sites contaminated with chlorinated ethylenes, such as tetrachloroethylene (PCE), trichloroethylene (TCE), *cis*-dichloroethylene (*cis*-DCE), and vinyl chloride (VC), whereas aerobic bioremediation is applicable to sites contaminated with benzene as well as VC (US EPA 2013b). Anaerobic degradation of chlorinated ethylenes is based on reductive dechlorination, in which chlorinated ethenes and hydrogen act as electron acceptors and an electron donor, respectively (Maymó-Gatell et al. 1997). A variety of anaerobic microbes that can degrade PCE or TCE have been identified. Among these, only *Dehalococcoides* sp., known as obligate anaerobic bacteria, can completely degrade chlorinated ethylenes to harmless ethylene, whereas biodegradation by other anaerobic bacteria terminates at TCE and/or *cis*-DCE (Gerritse et al. 1996; Maymó-Gatell et al. 1997; Holliger et al. 1998; Sung et al. 2006; Löffler et al. 2013). Apart from anaerobic biodegradation, aerobic biodegradation of chlorinated ethylenes except PCE has also been studied extensively because chlorinated ethylenes with fewer chlorine substituents undergo oxidative degradation more easily by either aerobic cometabolism or direct oxidation (Wilson and Wilson 1985; Nelson et al. 1988; Tsien et al. 1989; Elango et al. 2006). Aerobic degradation of chlorinated ethylenes is primarily based on cometabolism, a process for which cosubstrates such as methane, toluene, and/or phenol are required as growth substrates (Arp et al. 2001; Mattes et al. 2010); however, such cosubstrates are difficult to be introduced into contaminated sites, and the control of cometabolic processes is complex. Therefore, anaerobic degradation of chlorinated ethylenes is more widely used than aerobic degradation.

With regard to benzene, several bacteria, such as *Pseudomonas*, have been identified as aerobic degraders of benzene (Gibson et al. 1968; Fahy et al. 2006; Farhadian et al. 2008; Kim et al. 2008). Aerobic degradation of benzene is based on metabolic processes that degrade benzene to catechol (Gibson et al. 1968). Although benzene was once thought to persist in anaerobic environments, anaerobic benzene degradation has recently been observed under methanogenic, sulfate-reducing, iron-reducing, and nitrate-reducing conditions. Hydroxylation, methylation, and carboxylation are possible mechanisms of anaerobic benzene degradation (Foght 2008; Weelink et al. 2010; Vogt et al. 2011), and *Azoarcus* and *Desulfobacterales*- and *Coriobacteriaceae*-related bacteria are identified as anaerobic benzene degraders (Kasai et al. 2006; Noguchi et al. 2014). Although case studies on anaerobic biodegradation are available (e.g., Vogt et al. 2011; Borges et al. 2014), anaerobic benzene degraders have been identified in recent years, and anaerobic biodegradation of benzene requires two or more cooperative microbes (Vogt et al. 2011). This leads to a wider application of aerobic biodegradation of benzene.

Integrating anaerobic and aerobic biodegradation may be a promising approach for enhancing the efficiency of bioremediation and facilitating the complete biodegradation of multiple contaminants. A few studies have demonstrated the applicability of sequential anaerobic-aerobic biodegradation using a microbial consortium and an indigenous microbial community. Among the studies, biodegradation of a single or several similar chemicals, such as chlorinated ethylenes (Miller et al. 2007; Tiehm and Schmidt 2011), was discussed. They suggested integration of anaerobic biodegradation of PCE followed by aerobic biodegradation of *cis*-DCE and VC. More recently, Frascari et al. (2013) studied sequential biodegradation of chlorinated ethylenes along with trichloroethane and chloroform and found that TCE, *cis*-DCE, VC, trichloroethane, and chloroform were degraded under aerobic cometabolic conditions, whereas PCE and TCE were degraded under anaerobic conditions. Although chlorinated ethylenes and benzene are the most common VOCs in industrially contaminated sites, studies on sequential biodegradation of chlorinated ethylenes and benzene are still not available.

In this study, we investigated the biodegradation of multiple contaminants containing chlorinated ethylenes (PCE, TCE, *cis*-DCE, and VC), benzene, toluene, and dichloromethane (DCM), as these substances sometimes co-occur at contaminated sites in Japan. The objective was to explore the possibility of complete biodegradation of multiple VOCs through sequential degradation steps, including anaerobic degradation of chlorinated ethylenes to ethylene by obligately anaerobic bacteria (*Dehalococcoides* sp.), and aerobic degradation of benzene. Aerobic degradation of VC was not included, however, because we considered it an intermediate product of anaerobic degradation of chlorinated ethylenes. A key point of concern was, therefore, whether anaerobic bacteria that contribute to biodegradation of chlorinated ethylenes would survive exposure to oxidative conditions. To examine this, we performed laboratory feasibility tests under anaerobic or aerobic conditions, followed by biodegradation tests under alternating conditions (i.e., aerobic or anaerobic conditions, respectively). The effects of oxygen concentration and duration of oxygen exposure on anaerobic and aerobic biodegradation were examined. Microbial communities in the culture solutions at the end of the biodegradation tests were analyzed, and their possible roles in biodegradation are discussed.

## Materials and Methods

### Soil Sample Used as Source of Microorganisms

A soil sample taken from a site in Japan contaminated with multiple VOCs, specifically PCE, TCE, *cis*-DCE, VC, benzene, toluene, and DCM, was used as the source of microorganisms. Because of the presence of VC, an intermediate product of the anaerobic degradation of PCE, TCE, and *cis*-DCE to ethylene, we assumed that some useful bacteria, such as *Dehalococcoides* sp., might exist at this site (Mészáros et al. 2013). The upper aquifer, which was 10 m thick at this site, had been excavated and the lower aquitard, which was 8 m thick, was present at the bottom of the pit at the time of sampling. A clayey soil sample 5 cm in diameter and 30 cm in length was collected from the bottom surface of the pit with a stainless steel core sampler. The core was immediately packed in a vacuum bag with an oxygen-absorbing agent, A-500HS (I. S. O., Japan), and sealed with a vacuum sealer to maintain anaerobic conditions. The sample was kept cold (below 4 °C) in a portable ice chest during transport and stored in a refrigerator in the laboratory. The soil contained 0.3% total organic carbon and had a pH of 8.1. The soil particle size ranged from 0.2 to 415.9 μm, with an average of 21.5 μm.

### Strategy for Integrated Anaerobic-Aerobic Biodegradation Tests

**Fig. 1:**
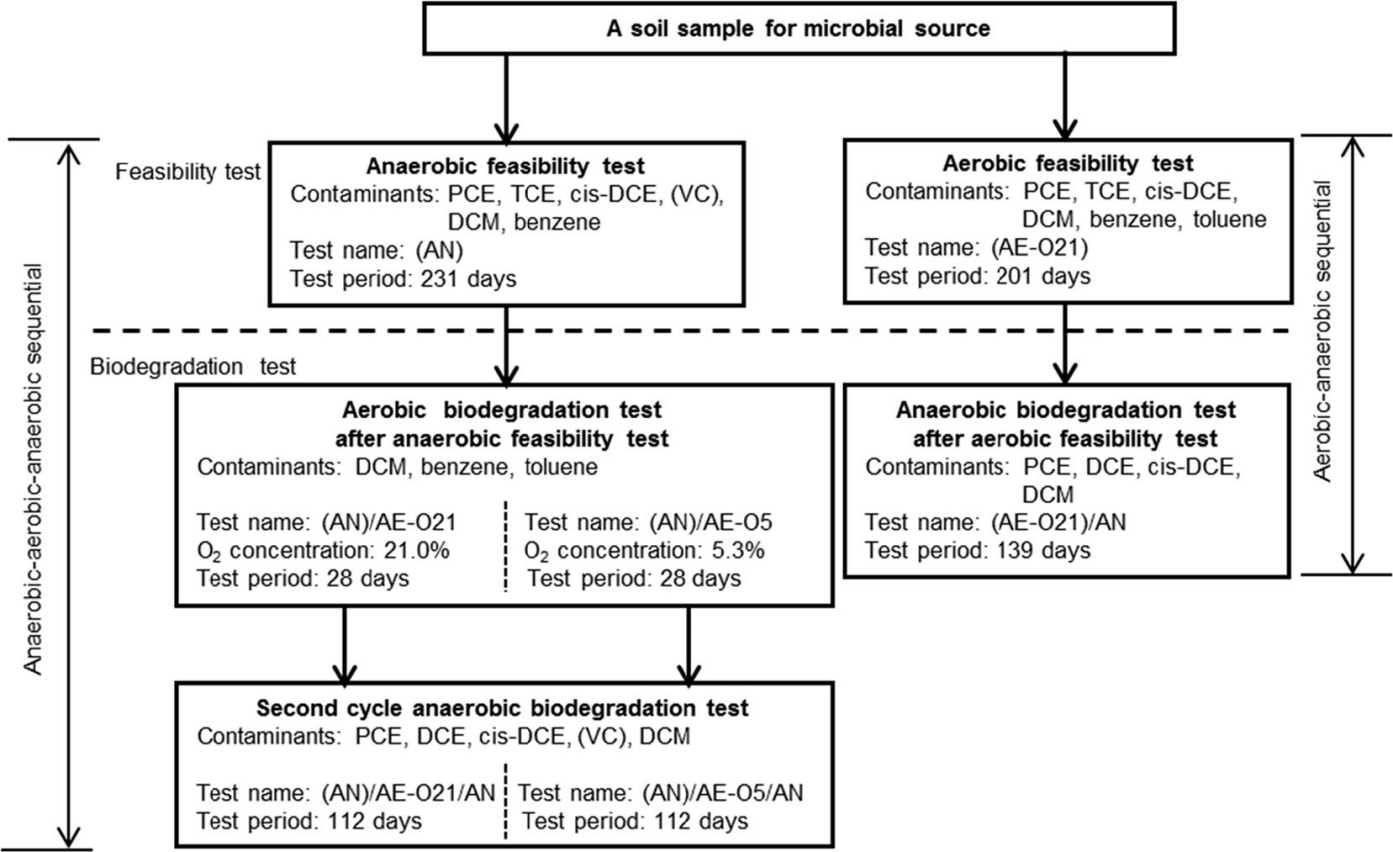
Framework of integrated anaerobic-aerobic biodegradation of multiple VOCs along with the major test conditions. Contaminants are the VOCs added to the test; (*VC*) indicates that vinyl chloride was a product of the degradation of other chlorinated ethylenes

To explore the possibility of complete biodegradation of the seven VOCs, we divided a series of sequential biodegradation tests into two steps (Fig. 1). The first step included feasibility tests of anaerobic and aerobic biodegradation of the VOCs. After examination of the results obtained from the feasibility tests, culture solutions from the first step were used as microbial sources for the second step; that is, cultures from the anaerobic and aerobic feasibility tests were transferred to biodegradation tests under alternate conditions (i.e., aerobic and anaerobic conditions, respectively). PCE, TCE, *cis*-DCE, VC, and DCM were added for anaerobic biodegradation tests, whereas benzene, toluene, and DCM were added for aerobic biodegradation tests. Oxygen concentration was changed to examine its effect on aerobic biodegradation and its potential influence on the second cycle of anaerobic biodegradation. For anaerobic biodegradation tests, the air in the headspace was replaced with N_2_ gas, and the concentration of oxygen was monitored to ensure anaerobic conditions.

### Feasibility Tests and Evaluation of Degradability of VOCs

Feasibility tests of the biodegradation of multiple VOCs were performed under both anaerobic and aerobic conditions, and the tests were designated (AN) and (AE-O21), respectively (Fig. 1). The wet soil sample, with a moisture content of 54.4%, was used as the source of microorganisms (concentration of bacterial 16S ribosomal RNA (rRNA) genes = 6.4 × 10^4^ copies/g) and mixed with a medium solution at a ratio of 1:2 (*w*/*w*). The medium solution was prepared according to the procedure described by Widdel and Pfennig (1981), but with different amounts of minerals and volume of vitamin solution. The medium solution comprised 1 L mineral solution, 0.10 mL trace elements solution, 0.40 mL vitamin solution, 0.10 mL Se + W solution, and 1.0 mL resazurin solution (Table 1). All reagents for the mineral medium were purchased from Wako Pure Chemical Industries (Japan). The mixture of the soil sample and the medium solution was agitated gently until settling of soil particles was no longer observed. Half of the mixture was aerated with N_2_ gas during agitation to maintain anaerobic conditions for anaerobic feasibility tests, whereas the other half was agitated in air for aerobic feasibility tests. The initial oxygen concentration in the headspace for aerobic tests was approximately 21.0%, equivalent to natural atmospheric concentrations. The suspension was dispensed into glass vial bottles with nominal volumes of 200 mL and actual volumes of 230 mL. Headspace volume for the aerobic tests was 140 mL.

**Table 1:** Concentrations of chemical compounds (g/L) in individual solutions used to prepare the medium solution

Solution	(g/L)	Solution	(g/L)
Mineral		Vitamin	
NaCl	1.00	Biotin	0.05
Na_2_SO_4_	0.11	*p*-Aminobenzoic acid	0.05
KH_2_PO_4_	0.14	Calcium pantothenate	0.05
NH_4_Cl	0.54	Pyridoxine-HCl	0.10
MgCl_2_·6H_2_O	0.20	Nicotinic acid	0.05
CaCl_2_	0.11	Thiamine-HCl	0.05
Trace element		Thioctic acid	0.05
FeCl_2_·4H_2_O	20.00	Folic acid	0.05
CoCl_2_·6H_2_O	2.40	Vitamin B_12_	0.05
MnCl_2_·4H_2_O	2.00	Riboflavin	0.05
ZnCl_2_	1.40	Se+W	
H_3_BO_4_	0.06	Na_2_SeO_3_	0.02
NiCl_2_·6H_2_O	0.24	Na_2_WO_4_·H_2_O	0.03
AlCl_3_·6H_2_O	0.24	Resazurin	
Na_2_MoO_4_·2H_2_O	0.24	Sodium resazurin	1.00
CuCl_2_·2H_2_O	0.02		

The bottles were tightly sealed with rubber stoppers and aluminum caps. For the anaerobic feasibility test, air in the headspace of the bottles was replaced with N_2_ gas. Sodium acetate, sodium lactate (Wako Pure Chemical Industries), and yeast extract (Kishida Chemical, Japan) were dissolved in ultrapure water at levels 100–200 times higher than the concentrations to be used for the tests within glass bottles, and the headspaces were replaced with N_2_; gas replacement was performed by first evacuating the headspace and then injecting N_2_ gas into the headspace. To ensure sufficient replacement, this procedure was repeated at least five times. An undiluted methanol solution (Wako Pure Chemical Industries) was used to avoid volatilization. Individual solutions were then added to sealed bottles with micro syringes until final concentrations were attained, which consisted of sodium acetate (0.5 g/L), sodium lactate (0.8 g/L), yeast extract (0.06 g/L), and methanol (0.4 g/L). For the aerobic feasibility test, air was allowed to remain in the headspace. Six VOCs, specifically PCE, TCE (Kanto Chemical, Japan), *cis*-DCE (Sigma-Aldrich, USA), benzene, toluene, and DCM (Wako Pure Chemical Industries), at concentrations of 30 mg/L in total volume (including soil particles) were added to each bottle; VC was not added because it could be generated if anaerobic biodegradation of PCE, TCE, and *cis*-DCE was successful. All bottles were placed upside down in an incubator set to 30 °C to avoid possible leakage of VOCs during long-term testing, and each feasibility test was carried out in duplicate to examine the reproducibility of individual tests. In addition, preliminary tests performed on autoclaved controls revealed that VOC concentrations remained more or less unchanged (data not shown), indicating that adsorption of VOCs was negligible for the soil being tested.

### Anaerobic and Aerobic Sequential Biodegradations

Following the success of the feasibility tests, aliquots of the culture solutions used in the anaerobic and aerobic feasibility tests were transferred to the medium solution as microbial sources, i.e., culture solutions at a ratio of 3:10 (*v*/*v*) for use in the subsequent aerobic and anaerobic biodegradation tests, respectively. The ratio was determined based on the data obtained from the preliminary trials (data not shown). Test bottles with nominal volumes of 100 mL and actual volumes of 120 mL were used in the sequential biodegradations, with a headspace volume of 94 mL. The composition of the medium solution was the same as that used in the feasibility tests. Headspace gases were replaced with N_2_ for anaerobic biodegradation and N_2_ + O_2_ for aerobic biodegradation. For aerobic biodegradation tests after the anaerobic feasibility test, headspace gases were first replaced with N_2_ gas, and then O_2_ gas (99.9% pure; GL Science, Japan) was injected until oxygen concentrations of 21.0% or 5.3% in the headspace of the bottles were attained. Considering ambient oxygen concentration in air, 21.0% was chosen as a typical condition for aerobic degradation. A lower oxygen concentration of 5.3% (one fourth of 21.0%) was also set for comparison purposes. The tests with 21.0% oxygen and 5.3% oxygen were designated (AN)/AE-O21 and (AN)/AE-O5, respectively, and the anaerobic biodegradation test after the aerobic feasibility test was designated (AE-O21)/AN. Concentrations of VOCs and electron donors in the biodegradation tests were the same as those in the feasibility tests. Similarly, these bottles were placed upside down in the dark at 30 °C, and each incubation condition was tested in duplicate. After the (AN)/AE-O21 and (AN)/AE-O5 tests, the culture solutions were transferred again, this time to anaerobic medium for further anaerobic biodegradation tests, which were designated (AN)/AE-O21/AN and (AN)/AE-O5/AN, respectively. The second cycle of anaerobic biodegradation was performed to examine the activity of anaerobic bacteria that had been exposed to oxygen.

### Analysis of VOCs and Oxygen

Concentrations of VOCs and ethylene in the vial bottles were analyzed using a GC-2014 gas chromatograph with a flame ionization detector (Shimadzu, Japan) and a GS-Q column (0.53 mm diameter, 30 m length; J&W Scientific, Agilent Technologies, USA). Gas concentrations in the headspace were converted to concentrations in the liquid phase under atmospheric pressure using Henry’s law constants (Mackay and Shiu 1981). For the biodegradation tests, detection limits of PCE, TCE, *cis*-DCE, VC, benzene, and toluene were 0.004 μmol/mL, whereas those of DCM and ethylene were 0.008 and 0.001 μmol/mL, respectively. Oxygen concentrations in the headspace were analyzed using a GC-8A equipped with a thermal conductivity detector (Shimadzu) and a Shincarbon column (3.0 mm diameter, 4 m length; Shinwa Chemical Industries, Japan). Dissolved oxygen (DO) concentrations in the liquid phase were converted from the oxygen concentrations in the headspace using Henry’s law constant disregarding the effects of soil particles.

### DNA Extraction

After the series of biodegradation tests, microbial cells together with soil particles were collected by centrifugation from the culture solutions in which VOCs had been successfully degraded. A 10-mL aliquot of each culture solution containing soil particles was centrifuged at 4640×*g* for 25 min, and DNA was extracted from the resulting 0.3–0.5-g pellets using a FastDNA® SPIN Kit for Soil (MP Biomedicals, USA). DNA extractions were performed in duplicate for the culture solutions tested under each condition. To increase the efficiency of DNA extraction, 20 mg of skim milk (Takada-Hoshino and Matsumoto 2004) was added to each pellet. In addition, the bead-beating method was adopted to facilitate isolation of DNA from a wide variety of microorganisms (de Lipthay et al. 2004; Verollet 2008: Yuan et al. 2012). The concentrations of DNA and microbial communities in the skim milk were analyzed in advance using DNA extracted from skim milk with the same kit to evaluate potential contamination. The DNA extracts from two bottles tested under the same conditions were mixed and used for microbiological analysis.

### Cloning of Bacterial 16S rRNA Libraries

Characterization of microbial communities in culture solutions was performed based on the analysis of the 16S rRNA gene, the most widely used genomic marker. Fragments of 16S rRNA genes were amplified from the DNA extracted from culture solutions using the universal primers 27f (5′-AGAGTTTGATCMTGGCTCAG-3′) and Bac1392R (5′-ACGGGCGGTGTGTAC-3′) (Lane 1991). The PCR conditions were optimized to minimize bias and artifacts by monitoring DNA amplification in preliminary trials to establish ideal parameters, such as the number of PCR cycles. These PCR products were ligated into a plasmid using the TOPO® TA Cloning® Kit for Sequencing (Invitrogen, USA), and the recombinant plasmids were used to transform HST08 competent cells (Takara Bio, Japan). Plasmid DNA was amplified by rolling cycle amplification using a TempliPhi kit (GE Healthcare, Japan) and sequenced with a 3730 xl DNA Analyzer (Applied Biosystems, USA) using primer 27f. Construction of bacterial clone libraries was based on the sequences of the bacterial 16S rRNA gene fragments, after the exclusion of chimeric clones with DECIPHER (Wright et al. 2012). Following the exclusion of chimeras, clones 63, 67, 61, and 52 obtained from the culture solutions in (AN)/AE-O21, (AN)/AE-O5, (AN)/AE-O21/AN, and (AN)/AE-O5/AN, respectively, were sequenced. The sequences were aligned using the software programs mothur (Schloss et al. 2009) and MEGA (Kumar et al. 2016). Sequences with >97% similarity were grouped into an operational taxonomic unit (OTU) with mothur software and the SILVA bacteria database (Pruesse et al. 2012). To determine the closest microorganisms, a representative sequence of each OTU was used for a BLAST search (Altschul et al. 1990).

## Results

### Degradability of VOCs in Feasibility Tests

All six VOCs were degraded in the feasibility tests (Table 2). Under anaerobic conditions, PCE, TCE, *cis*-DCE, and DCM were completely degraded in 231 days, whereas under aerobic conditions, benzene, toluene, and DCM were completely degraded in 159 days. VC was produced during the degradation of PCE, TCE, and *cis*-DCE and was also degraded. The oxygen concentration in the headspace was 14.7% when all of the benzene, toluene, and DCM were degraded, and DO concentration in the liquid phase could be converted to be 5.9 mg/L. Total oxygen decrease in the headspace (140 mL) was 15.8 mg, whereas the theoretical amount of oxygen required for aerobic biodegradation of the benzene and toluene added in one test bottle was calculated to be 16.7 mg based on the results reported by Wiedemeier et al. (1997) (3.08 and 3.13 mg of oxygen for every 1 mg of benzene and toluene, respectively). Dichlorination of DCM occurred without consumption of oxygen (Kohler-Staub and Leisinger 1985). These results indicate that the aerobic condition was sustained during the test. Based on these results, we proceeded with the sequential biodegradation tests.

**Table 2:** Preliminary findings of microbial degradation of volatile organic compounds (VOCs) in contaminated soil under anaerobic and aerobic conditions

VOCs	Preliminary tests	
	(AN)	(AE-O21)
PCE	+	−
TCE	+	−
*cis*-DCE	+	−
Benzene	−	+
Toluene	n.t.	+
DCM	+	+

*AN* preliminary anaerobic test, *AE-O21* preliminary aerobic test, *+* target VOC degraded, − target VOC not degraded, *n.t.* not tested

### Aerobic Biodegradation After Anaerobic Biodegradation

**Fig. 2:**
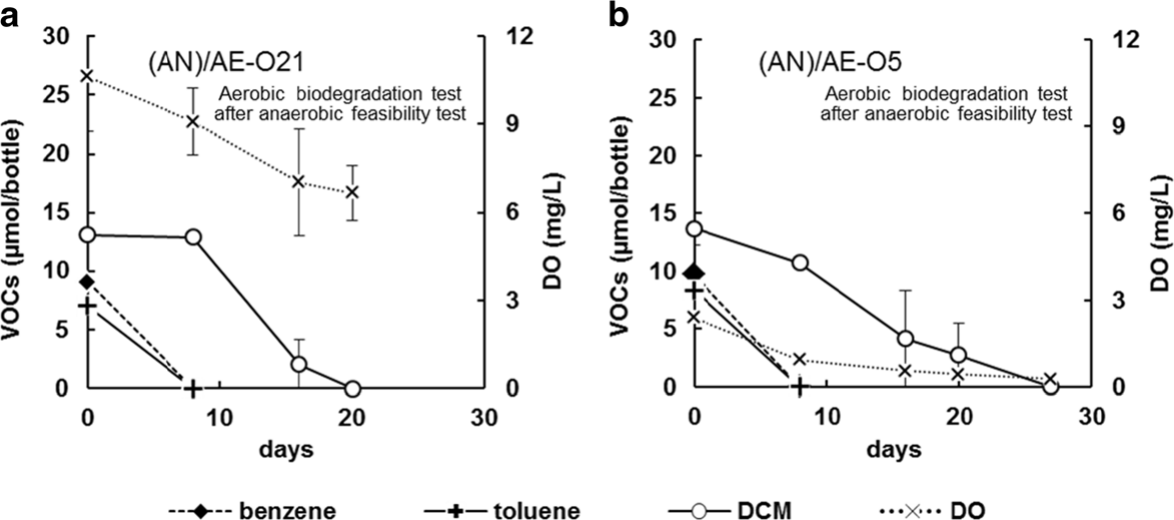
Aerobic degradation of VOCs over time, following preliminary anaerobic conditions. *Error bars* show the range of duplicate tests. Where *error bars* are not shown, the differences between duplicate values are smaller than the size of the *symbol*. Oxygen concentrations in the headspace of serum bottles were adjusted to **a** 21.0% and **b** 5.3% at the start of the aerobic incubation

Benzene, toluene, and DCM were completely degraded in the (AN)/AE-O21 and (AN)/AE-O5 tests (i.e., at both oxygen concentrations), with DCM degradation occurring after benzene and toluene degradation but within 20–27 days (Fig. 2). The difference in biodegradation of benzene and toluene was not observable within an analytical interval of 8 days; biodegradation of DCM showed a slight variation with higher speed under higher oxygen concentration.

Analysis of microbial communities from the (AN)/AE-O21 and (AN)/AE-O5 tests yielded 16 and 33 OTUs, respectively. Microbes from the skim milk accounted for less than 0.06% of those contained in culture solutions, indicating that DNA contamination from the skim milk was negligible. Microbial communities changed with oxygen concentration during the aerobic incubation (Table 3). Approximately 90% of the clones in (AN)/AE-O21 were classified in the phylum *Proteobacteria*, whereas 44.8% and 16.4% of the clones in (AN)/AE-O5 were classified as *Proteobacteria* and *Chloroflexi*, respectively. During the aerobic biodegradation, *Pseudomonas* was predominant in both (AN)/AE-O5 and (AN)/AE-O21. In (AN)/AE-O21, the OTU *Pseudomonas* 1 was as high as 31.7%, whereas it was not detected in (AN)/AE-O5. The OTU *Pseudomonas* 2 was detected in both (AN)/AE-O21 and (AN)/AE-O5. In addition to *Pseudomonas*, we detected *Geobacter*, *Hyphomicrobium*, *Acidovorax*, and *Dehalococcoides* in both (AN)/AE-O21 and (AN)/AE-O5, and 12.7% of the clones in (AN)/AE-O21 were *Sulfuricurvum*.

**Table 3:** GenBank matches to cloned bacterial 16S rRNA gene fragments in tests (AN)/AE-O21, (AN)/AE-O5, (AN)/AE-O21/AN, and (AN)/AE-O5/AN

Closest GenBank match		Frequency of clones in each library (%)			
Names of phylum and OTU	Closest isolate [Accession no.] (% identity)	(AN)/AE-O21	(AN)/AE-O5	(AN)/AE-O21/AN	(AN)/AE-O5/AN
*Bacteroidetes*					
*Paludibacter*-like	*Paludibacter propionicigenes* [AB910740] (94)		5 (7.5)		
Other *Bacteroidetes*			2 (3.0)	3 (4.9)	1 (1.9)
*Chloroflexi*					
*Dehalococcoides*	*Dehalococcoides mccartyi* GT [CP001924] (100)	1 (1.6)	2 (3.0)	7 (11.5)	8 (15.4)
*Leptolinea*-like 1	*Leptolinea tardivitalis* YMTK-2 [NR_040971] (90)		3 (4.5)	8 (13.1)	1 (1.9)
*Leptolinea*-like 2	*Leptolinea tardivitalis* YMTK-2 [NR_040971] (91)		2 (3.0)	1 (1.6)	1 (1.9)
Other *Chloroflexi*		2 (3.2)	4 (6.0)	6 (9.8)	2 (3.8)
*Firmicutes*					
*Trichococcus*	*Trichococcus patagoniensis* AB-190 [KF817793] (100)	1 (1.6)	1 (1.5)	7 (11.5)	9 (17.3)
*Acetobacterium*	*Acetobacterium malicum* DSM4132 [NR_026326] (99)				15 (28.8)
*Acidaminobacter*	*Acidaminobacter* sp. CJ5 [GU570195] (99)		3 (4.5)		1 (1.9)
*Clostridium*-like 1	*Clostridium* sp. 6-44 [AB596885] (96)			2 (3.3)	
*Clostridium*-like 2	*Clostridium acidisoli* [AJ237756] (93)				2 (3.8)
*Youngiibacter*	*Youngiibacter multivorans* [AB910755] (99)		2 (3.0)		
Other *Firmicutes*			4 (6.0)	6 (9.8)	3 (5.8)
*Proteobacteria*					
*Pseudomonas* 1	*Pseudomonas* sp. JN18_A17_R [DQ168645] (100)	20 (31.7)		16 (26.2)	1 (1.9)
*Pseudomonas* 2	*Pseudomonas stutzeri* DQ-1 [KC460328] (100)	16 (25.4)	10 (14.9)		
*Geobacter* 1	*Geobacter* sp. IFRC128 [HQ687068] (97)		4 (6.0)		
*Geobacter*-like 1	*Geobacter* sp. CdA-2. [Y19190] (96)	5 (7.9)	5 (7.5)		
*Geobacter*-like 2	*Geobacter* sp. CdA-2. [Y19190] (94)		1 (1.5)		
*Sulfuricurvum*	*Sulfuricurvum kujiense* YK-2 [AB080643] (98)	8 (12.7)			
*Hyphomicrobium*	*Hyphomicrobium* sp. LAT3 [AY934489] (99)	2 (3.2)			
*Acidovorax*	*Acidovorax* sp. C34 [JX177702] (100)	2 (3.2)	1 (1.5)		
Other *Proteobacteria*		3 (4.8)	7 (10.4)	2 (3.3)	1 (1.9)
Others		1 (1.6)	4 (6.0)	1 (1.6)	4 (7.7)
Unclassified		2 (3.2)	5 (7.5)	2 (3.3)	3 (5.8)

### Anaerobic Biodegradation After Sequential Anaerobic and Aerobic Biodegradations

**Fig. 3:**
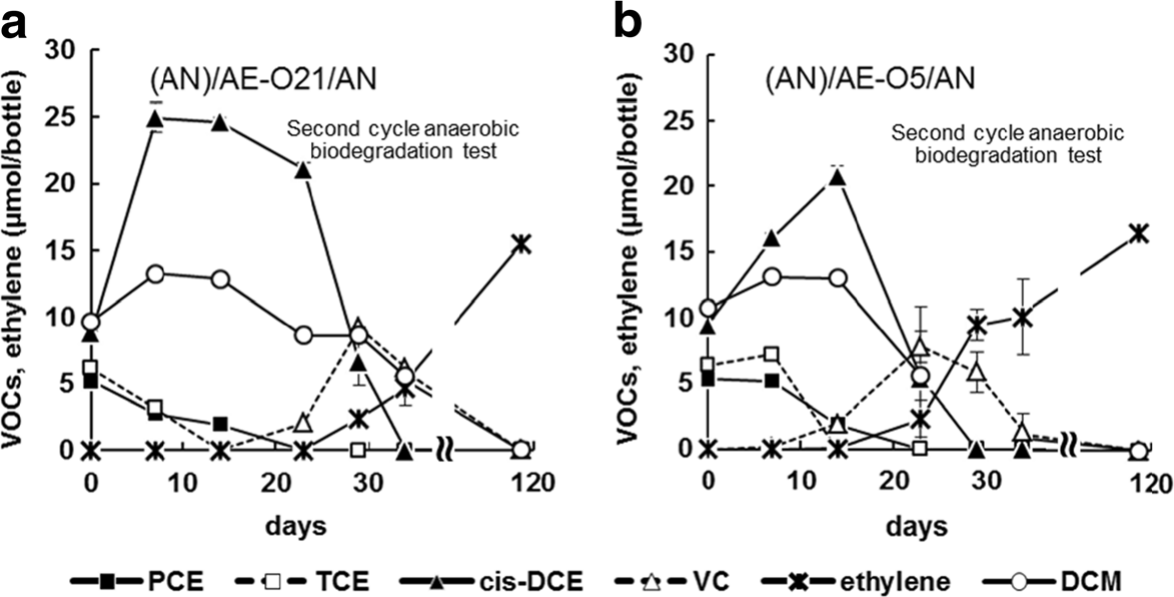
Anaerobic degradation of VOCs over time, following preliminary anaerobic and aerobic conditions. Oxygen concentrations in the headspace of serum bottles were adjusted to **a** 21.0% and **b** 5.3% at the start of the preliminary aerobic incubation. *Error bar*s show the range of duplicate tests. Where *error bars* are not shown, the differences between duplicate values are smaller than the size of the *symbol*

PCE, TCE, *cis*-DCE, VC, and DCM were degraded in the (AN)/AE-O21/AN and (AN)/AE-O5/AN tests, which were derived from the culture solutions previously used for sequential anaerobic and aerobic biodegradations (Fig. 3). All of the added contaminants were degraded to levels below the detection limits within 112 days. PCE and TCE were almost completely degraded after 14 days in the (AN)/AE-O21/AN and (AN)/AE-O5/AN tests, with *cis*-DCE and VC following sequentially. In both the (AN)/AE-O21/AN and (AN)/AE-O5/AN tests, previously exposed to oxygen for 28 days during aerobic degradation, PCE was stably dechlorinated to ethylene via VC. Degradation of *cis*-DCE started earlier in (AN)/AE-O5/AN (day 14) than in (AN)/AE-O21/AN (day 23).

Clonal analysis showed that the culture solutions tested in the second cycle of anaerobic biodegradation, (AN)/AE-O21/AN and (AN)/AE-O5/AN, contained *Dehalococcoides* clones at 11.5% and 15.4%, respectively (Table 3). In (AN)/AE-O5/AN, *Acetobacterium* was detected as high as 28.8%, whereas it was not detected in (AN)/AE-O21/AN.

### Anaerobic Biodegradation After Aerobic Biodegradation for 201 Days

**Fig. 4:**
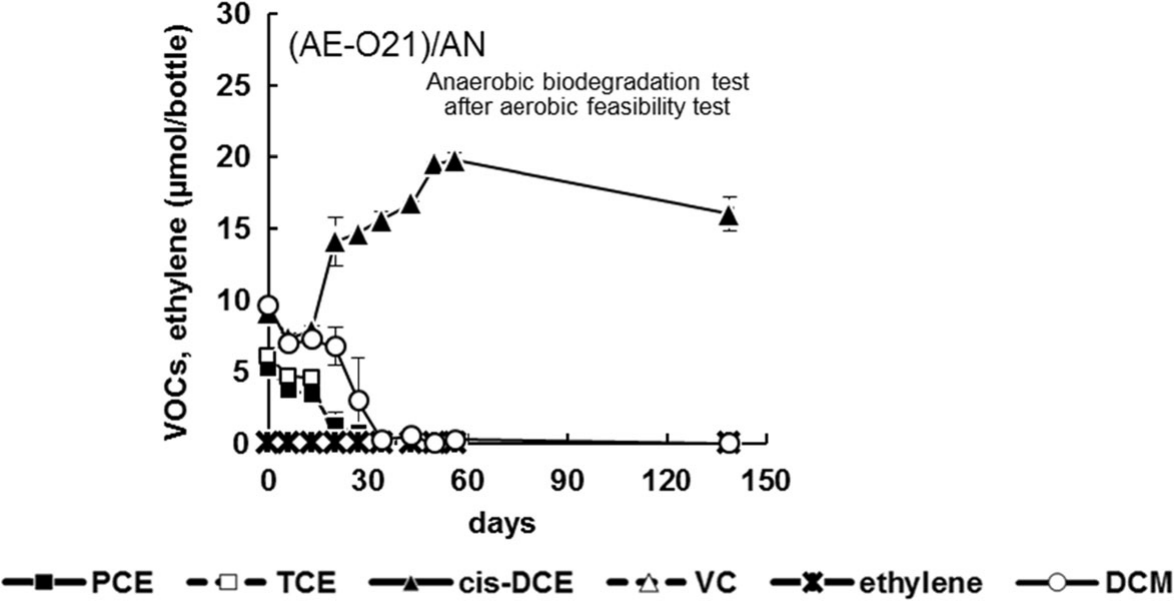
Anaerobic degradation of VOCs over time, following preliminary aerobic conditions. *Error bars* show the range of duplicate tests. Where *error bars* are not shown, the differences between duplicate values are smaller than the size of the *symbol*

PCE, TCE, and DCM were degraded in the (AE-O21)/AN test, which was derived from the culture solution that was previously used for aerobic biodegradation (Fig. 4). In contrast, the amount of *cis*-DCE increased after 20 days and remained constant after 130 days. The daughter product VC, which was produced in the (AN), (AN)/AE-O21/AN, and (AN)/AE-O5/AN tests, was absent in the (AE-O21)/AN test. We concluded that the (AE-O21)/AN test was inadequate for the degradation of all seven VOCs and (AE-O21)/AN was not further transferred for aerobic biodegradation tests.

## Discussion

### Possibility of Complete Biodegradation of Multiple VOCs

Benzene, toluene, and DCM were completely degraded under aerobic conditions after anaerobic biodegradation of PCE, TCE, *cis*-DCE, and VC (Table 2 and Fig. 2). These results suggest that sequential anaerobic and aerobic biodegradation can eliminate the following seven pollutants: PCE, TCE, *cis*-DCE, VC, benzene, toluene, and DCM. In common engineering practice, enhanced anaerobic biodegradation can be achieved by injecting lactate, molasses, hydrogen release compound, and vegetable oils as electron donors (Parsons 2004). On the other hand, engineering practices such as injection of an oxygen release compound (Chapman et al. 1997), pulsed air sparging (Yang et al. 2005), and electrolytic oxygen generation (Franz et al. 2002) have been applied at remediation sites to change oxygen conditions in situ to create an aerobic environment. Integrated anaerobic and aerobic biodegradation may be of practical use with these engineering techniques.

### Microbes Involved in Aerobic Biodegradation

In aerobic biodegradation after anaerobic biodegradation, *Pseudomonas*, *Acidovorax*, and *Hyphomicrobium* might be involved in the degradation of benzene, toluene, and DCM. The OTU *Pseudomonas* 2 was highly similar to *Pseudomonas stutzeri* DQ-1 (Table 3). Oxygenase produced by a strain of *P. stutzeri* was found to degrade benzene and toluene (Bertoni et al. 1996), suggesting that *Pseudomonas* 2 may have been involved in the degradation of these compounds in our study. The closest isolate to OTU *Acidovorax*, *Acidovorax* sp. C34 (Table 3), degrades an aromatic compound phenol, an intermediate product of aerobic biodegradation of benzene, using phenol hydroxylase and catechol 2,3-dioxygenase. In addition, Fahy et al. (2006) considered *Acidovorax* spp. to be microbes capable of aerobically degrading benzene by using benzene as a possible source of both carbon and energy; therefore, *Acidovorax* may have been involved in the aerobic degradation of benzene in our study. Previous studies have indicated that some *Hyphomicrobium* strains degrade DCM through dehalogenation metabolism (Muller et al. 2011). The closest isolate to OTU *Hyphomicrobium* in this study, *Hyphomicrobium* sp. LAT3 (Table 3), uses methyl chloride (Borodina et al. 2005). Therefore, the OTU *Hyphomicrobium* might be involved in the degradation of DCM. Microbes involved in aerobic biodegradation of benzene, toluene, and DCM may survive even long-term exposure to anaerobic conditions; the preliminary anaerobic feasibility test in this study lasted for 231 days. In addition, the soil sample was originally taken from the site under anaerobic condition and was packed and stored under anaerobic condition prior to the anaerobic feasibility tests. This suggests that aerobic microbes can survive for long periods under anaerobic conditions. It is possible that aerobic microbes such as *Pseudomonas* and *Hyphomicrobium* could survive by using alternative electron acceptors, such as nitrate, when oxygen is depleted (Lalucat et al. 2006; Martineau et al. 2015). In this study, the ammonium chloride that was added to the medium solution could be oxidized to nitrate during the aerobic degradation process. In practical applications, therefore, less care is required to ensure the survival of aerobic microbes under anaerobic conditions when alternative electron acceptors are present.

### Microbes Involved in Anaerobic Biodegradation

In anaerobic biodegradation after sequential anaerobic and aerobic biodegradations, (AN)/AE-O21/AN and (AN)/AE-O5/AN contained OTU *Dehalococcoides* at high percentages (Table 3). Although (AN)/AE-O21/AN and (AN)/AE-O5/AN were exposed to different oxygen concentrations during the previous aerobic biodegradation tests, the differences in their percentages were small. These results suggest that some *Dehalococcoides* may have survived or became active even after exposure to aerobic conditions when the concentration of dissolved oxygen in culture solutions varied between 0.2 and 10.0 mg/L (Fig. 2), which contributed to dechlorination under anaerobic conditions, although oxygen has been thought to inhibit the dechlorination of VC by *Dehalococcoides* sp. (Richardson et al. 2002; Amos et al. 2008). In addition, the soil particles in the test bottles may function as a buffer and protect some *Dehalococcoides* from direct exposure to oxygen. Under in situ conditions, the ratio of soil to water is much higher than that used in laboratory tests, depending on geological conditions. We suggest that the oxygen tolerance of *Dehalococcoides* and/or the presence of soil particles in the biodegradation environment may have contributed to the stable habitation and activation of *Dehalococcoides* populations. *Acetobacterium*, which is known to produce acetic acid that can be used as a carbon source for *Dehalococcoides* (Kittelmann and Friedrich 2008), was detected in (AN)/AE-O5/AN (Table 3). This could potentially be the reason for the earlier initiation of *cis*-DCE degradation (Fig. 3). To date, only two studies have reported the isolation of microbes that anaerobically degrade DCM: *Dehalobacterium formicoaceticum* DMC (Mägli et al. 1996) and *Dehalobacter* sp. strains (Justica-Leon et al. 2012). Two clones from (AN)/AE-O21/AN that were classified into other *Firmicutes* had 94.5% similarity with *D. formicoaceticum* DMC. No clones with similarities over 90% with *Dehalobacter* sp. strains were identified from the tested samples. Since a similarity score lower than 97% generally represents a different species (Stackebrandt and Goebel 1994), we suggest the presence of yet-unknown microbes that anaerobically degrade DCM. Further studies on the isolation and identification of DCM degraders under anaerobic conditions are necessary for a deeper understanding of the mechanisms involved in anaerobic biodegradation of DCM.

### Tolerance of *Dehalococcoides* to Oxygen

Degradation of *cis*-DCE was not observed in the (AE-O21)/AN test, which involved exposure to oxygen for 201 days during preliminary aerobic biodegradation (Fig. 4). However, in the (AN)/AE-O21/AN and (AN)/AE-O5/AN tests, which involved exposure to oxygen for 28 days during the previous aerobic degradation, PCE was stably dechlorinated to ethylene via VC (Fig. 3). The results indicate that the length of the aerobic period affects the viability of anaerobic bacteria and, thus, subsequent anaerobic biodegradation. In general, the higher the oxygen concentration and the longer the exposure time, the more severe is the damage to anaerobic bacteria. Oxygen will be reduced to superoxide and hydrogen peroxide within microbial cells and damage DNA, proteins, membranes, and/or enzymatic activity of microbial cells (Imaly 2002). Marschall et al. (1993) reported that *Desulfovibrio desulfuricans* Essex lost its viability within 3 and 12 days when it was exposed to oxygen concentrations of 5% and 1%, respectively. Such quantitative information is still lacking for *Dehalococcoides*. Further studies on the effects of oxygen concentration and exposure period on the viability of anaerobic bacteria, such as *Dehalococcoides* strains, that contribute to degradation of chlorinated ethylenes are fundamentally necessary. The results of this study illustrate that the degradation of *cis*-DCE by *Dehalococcoides* may be influenced by the duration of oxygen exposure (initial oxygen concentration of 21.0%, corresponding to converted DO concentration of 8.4 mg/L), with *Dehalococcoides* dying or losing activity following incubation under aerobic conditions for 201 days. Since the length of aerobic period affects the viability of anaerobic bacteria, the time period for aerobic biodegradation in the sequential process becomes a key issue. It is advisable to maintain aerobic conditions as short as possible while ensuring benzene, toluene, and DCM are degraded to acceptable levels.

## Conclusions

Integration of anaerobic and aerobic biodegradations resulted in the degradation of seven VOCs: PCE, TCE, *cis*-DCE, VC, benzene, toluene, and DCM. PCE, TCE, *cis*-DCE, VC, and DCM with initial concentration of each VOC less than 30 mg/L were degraded under anaerobic conditions. Benzene and toluene were degraded within 8 days, and DCM was degraded within 20–27 days when concentrations were lower than 30 mg/L under aerobic conditions and initial oxygen concentrations in the headspaces of test bottles were set to 5.3% and 21.0%, corresponding to the converted DO concentrations in the liquid phase of 2.1 and 8.5 mg/L, respectively. The length of the aerobic period affected subsequent anaerobic biodegradation. *Dehalococcoides* sp. survived aerobic conditions for 28 days and could be activated during subsequent anaerobic biodegradation. However, degradation of *cis*-DCE was suppressed after oxygen exposure for more than 201 days. Anaerobic degradation of DCM after oxygen exposure was complete. Yet-unknown microbes may contribute to the anaerobic degradation of DCM; further studies on the isolation and identification of DCM degraders under anaerobic conditions are necessary for a deeper understanding of the mechanisms involved. The findings of this study may provide a scientific and practical basis for the complete bioremediation of multiple contaminants in situ and a subject for further exploration.
